# Addressing the concept of total pain in palliative care: A postcolonial South African hospice view

**DOI:** 10.1017/S1478951524001123

**Published:** 2025-01-28

**Authors:** Michelle Petersen-Damon, Leslie Swartz

**Affiliations:** Department of Psychology, Stellenbosch University, Stellenbosch, South Africa

**Keywords:** Palliative care, hospice, diversity, holism, postcolonial, South Africa

## Abstract

**Objectives:**

Palliative care, which was formally established in the Global North, is now recognized globally as part of health care. As part of a larger study, we were interested in how decision-makers at a leading hospice in South Africa understood the changing local context and its influence on the delivery of services. We were interested in how the concept of “total pain,” as outlined by Saunders, applies in a very unequal and under-resourced society in the shadow of a long, oppressive colonial, and apartheid past.

**Methods:**

We conducted face-to-face semi-structured interviews with 12 staff at St Luke’s Combined Hospices in Cape Town, South Africa, and analyzed the data following Braun and Clarke’s thematic analysis approach.

**Results:**

Four major themes emerged from the data. First, St Luke’s has faced the challenge of serving a larger and far more diverse population than it had under apartheid. Second, the organization has undergone a process of rethinking holism and holistic services offered to palliative care patients in this context. Third, diversity and cultural sensitivity are key to how services are offered, and finally, the concept of “total pain” in this context is linked to questions of power and empowerment.

**Significance of results:**

This study is small and situated within a particular context, and it is clear that more data are needed. Nevertheless, the study shows that considering the Global South and postcolonial context is important for thinking about total pain and a global system of palliative care which is sensitive to the majority world context.

## Introduction

Palliative care is now globally recognized as a key component of health care (Brennan 2007). It is also widely understood that palliative care practice will be influenced by local practices and resources. In this regard, when taking a global perspective it is important to recognize that palliative care as a field has its roots in the work of Dame Cecily Saunders in the United Kingdom (UK), a high-income European country, and thus a country dissimilar in many respects from most countries in the world, these being low- and middle-income countries (Abu-Odah et al. 2020; Clark et al. 2007; Mason et al. 2020).

In formulating the basis of palliative care, in the 1940s Saunders introduced the concept of “total pain” (Timm 2023). The concept of total pain encompasses the person in totality and focusses on the spiritual, physical, social, and psychological aspects of each individual (Brant 2017; Mehta and Chan 2008; Ong and Forbes 2005). The concept and experience of total pain have been extensively researched (Greenstreet 2001; Krawczyk et al. 2018; Wood 2022), and a key feature of the research extends beyond total pain as an holistic concept, to pain as contextual – both the experience of pain, and the best way to provide palliative care, will differ contextually (Grant et al. 2011; Mahilall and Swartz 2021). In this article, we consider the provision of palliative care in South Africa, an upper middle-income country with a history of colonialism and apartheid, and ongoing inequality consequent to centuries of oppression and exclusion. It is significant to note that the field of palliative care in South Africa was officially established as a recognized area of health care relatively recently (Republic of South Africa 2017).

The first formal hospice in the Global South was established in Harare, Zimbabwe, a neighboring country to South Africa, in 1979 (Zvakawapano 2019). In Africa, the need for palliative care services remains ongoing where an overwhelming majority of Africans currently endure progressive, life-limiting illnesses, yet access to culturally appropriate holistic palliative care (including effective pain management) is simply not available (African Palliative Care Association 2023). Palliative care and pain management are among the most critical challenges faced by resource-limited settings in Africa.

Locally, in South Africa, palliative care is provided primarily by nongovernmental organizations (NGOs) (Drenth et al. 2017; Gwyther et al. 2018; O’Brien et al. 2019). One such NGO is St Luke’s Combined Hospices (SLCH), a well-known hospice based in Cape Town, South Africa. SLCH provides an extensive palliative care program that spans across various physical settings. These settings include a dedicated inpatient unit, community-based day hospices^1^ where individuals with terminal illnesses, but not in their final stages of life, can gather and receive care during the day, as well as the provision of care in patients’ own homes. Care is provided by a team of skilled professionals (social workers, professional nurses, and spiritual bereavement care workers) and volunteers. The holistic needs of patients and families are addressed, with the focus of hospice services being on providing quality care for those who are dying, and a key component is that of end-of-life care to ensure that the individual transitions to their death with dignity. SLCH has come a long way since its inception some 43 years ago. The Cape Town-based hospice was founded in 1980 (a year after the Zimbabwean hospice was established) and registered as a nonprofit company, and is recognized as a center of excellence that is dedicated to palliative care services and serves a total of over 600 patients daily across Cape Town.

Looking into the history of the organization, SLCH was started in the apartheid era by a small group of Christian-based volunteers from affluent areas, largely made up of Whites^2^ who had the notion of giving back to society. Volunteers were typically relatively wealthy, privileged retirees with time availability and a commitment to giving back to society. At the time, however, the voluntary services offered by the organization were available to a select few who were privileged enough to have the means to access these services. Services were based in what at that time was legally designated a “White” area, and, for many South Africans, transport costs to that area made the service relatively inaccessible.

South Africa became a democracy in 1994. As with services in many areas of the country, prior to the advent of democracy, SLCH began to expand its services to underserved areas where the social contexts differed from that of the more affluent, former White areas. SLCH based its services on UK models, but it became clear that, given the diversity of South Africa in terms of race, class, religion and language, coupled with the ongoing effects of centuries of oppression and exclusion, SLCH would have to evolve in order to address the pain continuum in the South African context.

Despite South Africa having been a democracy for 30 years, the country remains one of the most unequal societies in the world, with very high rates of violence and intergroup mistrust. SLCH has had to take this into account in providing community-based palliative care services. The communities now served by SLCH are very diverse, including both affluent suburbs and the less resourced and marginalized communities within the greater Cape Town area of South Africa. The organization has 6 community day hospices based in 6 different communities, and these facilities can be seen as a one-stop shop where patients attending have access to holistic services, including psychosocial, medical and spiritual/bereavement interventions, but also focusing on social exclusion within the diverse areas. Currently, SLCH provides a comprehensive palliative care service that extends to various physical settings in the form of a specialized inpatient unit, community-based day hospices as well as care in the community within the comfort of the patients’ own homes. SLCH has staff fulfilling various profiles, from administrative support to the clinical, patient-facing team.

In the context of total pain, social pain is particularly prominent in South Africa, given the country’s history and current troubled present. Social pain may be affected by a variety of factors. Key issues which need consideration when thinking about social pain in South Africa include, but are not limited to the following:

- **Cultural diversity and cultural sensitivities**: With the country’s history, South Africa is known for its diversity in the cultural arena, with a rich mix of ethnicity, languages, areas, religions, and traditions. A significant body of research, particularly in South Africa, has delved into the topic of cultural diversity within health care settings and its effects on the quality of care (Chandramohan and Raisuyah 2016; Matthews and Van Wyk 2018; Swartz 1991). In the context of palliative care specifically, cultural sensitivity may require acknowledging and integrating traditional and religious healing practices, spiritual beliefs, and cultural rituals into the care of patients at the end of life.
  - **Stigma and discrimination**: There is a long history of discrimination against people of color in South Africa, and of lack of provision of services, partly on the basis of this discrimination (Swartz 1996). Furthermore, there is often a stigma attached to death, dying, and serious illness in many societies and communities. Though there is evidence that there is a benefit to early palliative care, which is endorsed by oncology societies, negative connotations persist regarding palliative care (Zimmermann et al. 2016) for patients, caregivers, and the public. This may be exacerbated in a country like South Africa where palliative care services might be viewed as appropriate only to the needs of White people, as traditionally this was the group receiving palliative care.
  - **Socioeconomic inequalities**: South Africa has significant socioeconomic disparities, which is the legacy of colonialism, and this remains the reality in post-colonialism in the country. Despite the efforts of SLCH, patients from less affluent or marginalized communities continue to experience challenges in access to quality palliative care services due to financial limitations and geographical barriers. Tertiary hospitals offering the most advanced medical services for many terminal conditions remain located in historically White areas, and safe, affordable transport is a challenge for many.

Addressing social pain in palliative care in the South African context requires a comprehensive, holistic, and focused approach which considers and addresses cultural diversity and sensitivity, linguistic barriers, and a collaborative relationship among the palliative care team, patients, families, and the broader community. Further, this approach should be cognizant of the impact of the social context and, importantly, the historical context within which social pain in palliative care is understood.

Given the changing context of palliative care in South Africa, and as part of a larger study of organizational change, we were interested in how the management team at SCLH and service providers view palliative care services in contemporary South Africa, especially in light of the major social challenges facing the country, challenges which are not as marked in Global North contexts.

## Methods

We approached all the policymakers at SLCH and asked them to participate in this study, and all agreed, as did selected service providers in community-based positions. Ethical clearance for the study was received from Stellenbosch University and from SLCH itself.

We conducted one-on-one interviews with the various team members working in management and senior service positions in communities, as well as with policy-makers (managers), to ascertain their opinions and suggestions regarding palliative care service delivery in South Africa, with a particular emphasis on total pain. Table 1 provides the biographical data of the 9 participants who participated in the study.

**Table 1.** Biographical data of participants who participated in the study

Participant number	Race: Black (B), colored (C), other (O), White (W); gender: female (F), male (M)	Designation at SLCH	Number of years employed at SLCH in the designation
1	O (F)	Management	7
2	W (F)	Management	4
3	W (F)	Management	5
4	W (F)	Management	26
5	W (F)	Management	1 year 6 months
6	C (M)	Spiritual bereavement	7
7	C (F)	Social worker	5
8	B (F)	Social worker	1 year 3 months
9	B (F)	Professional nurse	8
10	B (M)	Professional nurse	1
11	C (F)	Social worker	6 months
12	B (M)	Spiritual bereavement	12

A qualitative method was employed with in-depth interviews. This facilitated the gathering of data which were detailed and descriptive in nature (Hollway and Jefferson 2013). The duration of the individual interviews with each participant ranged between 45 and 60 minutes.

The one-on-one interviews were structured with open-ended questions, allowing the participants to give their own opinion on the discussion topic. The interviews were recorded and transcribed, and each participant was allocated a code for anonymity and protection of personal information. Participant responses were transcribed using thematic analysis, which Braun and Clarke (2006) define as a qualitative research method involving the gathering of data for the purpose of identifying, analyzing, and reporting patterns. The data were then coded and reviewed for emerging themes.

## Results

The study identified 4 prevalent themes, which are listed below, and each theme is discussed in turn:

1. A larger and more diverse palliative care recipient population.
2. Rethinking holism and holistic services to palliative care patients.
3. Diversity and culturable sensitivity in palliative care.
4. Total pain and empowerment.

### Theme 1: A larger and more diverse population

The shift in SLCH from effectively a “Whites only” service to a much wider service was prominent in the responses:

There was a shift from the conventional palliative care going home into the in-patient unit now extended and was largely White and largely upper class. (Participant #1)

I think in the beginning years the hospice was just serving people who were financially better off than others. … the service was attended only on the western side [the more affluent side]. (Participant #6)

… for what I understand, St Luke’s used to provide services only to the White areas at the time [before evolvement], it was not in our [non-White] communities. … the organization, yes, it changed now. It includes everyone now, like our areas [previously known as Black areas], even the so-called Colored areas, they are also benefitting, so yeah, it’s [hospice] everywhere. (Participant #7)

### Theme 2: Rethinking holism and holistic services to palliative care patients

It was noteworthy that, along with mentioning a wider reach of services in terms of population, participants commonly linked this to a more holistic approach to care:

… to do away with the different constructs of palliative care operating in little pockets … we want palliative care to be interdisciplinary palliative care, to be mirrored in all aspects of service delivery, community-based interdisciplinary palliative care, to in-patient unit interdisciplinary palliative care, to the referral’s office interdisciplinary palliative care. So that is the model we are propagating. … there will be understanding that a human being requires multiple services almost simultaneously. (Participant #1)

So, you can’t therefore say, ok now, I’m doing this medical intervention and tomorrow the social worker is going to come. It doesn’t matter you are weeping now and to hang on with your weeping, tomorrow the social worker will come and sort it out. (Participant # 1)

What was also clear was that for SLCH, holistic or comprehensive care has to be thought about in the context of a lack of other social services in communities, with the role of SLCH often going beyond the confines of focusing on issues of death and dying:

I think social work is very important because each and every house, even if there’s someone sick, there’s always [other] … big problems. (Participant #8)

Psychosocial needs are prevalent, and if SLCH does not render a generalized social work service to address these needs, they will not be addressed, as services from other organizations and state departments are often not available or accessible.

Participants reported that users of SLCH commented on changes in their households not directly related to palliative care, as narrowly understood:

… ever since you guys [SLCH] came, things have changed. My life was controlled by the children … and so the children are supporting me. (Participant #8)

The view of another participant supported the holistic services provided to patients across some of the 5 areas by the interdisciplinary team, with particular emphasis on the resources available in communities. The respondent alluded to the holistic services and the various role-players by stating the following:

… resources may be different [in the various communities] but it still covers everything that you require in any interdisciplinary team, which is looking after the patient from a clinical or a medical point of view, … the psychosocial side which is where the social workers come in and the spiritual and bereavement cases. Each of those areas [the areas included in the study] attend to not just the patient, attend to family members before, during and after the event [of death]. (Participant #2)

A key issue for many service users is lack of finances, and problems with access to social cash transfers:

Mostly the [disability] grant is not enough … the grant, and specifically SASSA [South African Social Security Agency] … there’s often a struggle to get the services from SASSA. They [SASSA] don’t understand if our patient is not in receipt [of a social security or support grant as financial assistance from the government/state] so … the people [patients] must go and stand, otherwise some people [SASSA officials] are rude. (Participant #8)

In the context such as that described above, the SLCH social worker, though focused primarily on palliative care, must follow up to facilitate access to social grants.

Other basic needs not commonly viewed as part of palliative care are prominent, especially in the most deprived areas:

Ok, I would say that the basic needs in XXX [an historically Black township with a large number of informal dwellings], it’s always a need for something to eat … like it’s more important for them [patients in XXX], unlike people in YYY [a more affluent area] … maybe for them it’s just family issues maybe, but for our people [in XXX], first thing, poverty is a problem. (Participant #8)

### Theme 3: Diversity and culturable sensitivity in palliative care

Participants noted that working with patients requires understanding and respect, and sensitivity across divides:

I think that culture must be respected … must be allowed to exist. We should take into account people that they [the SLCH team] serve comes from cultures. I do think that we don’t know enough about each other’s cultures and that if we work more across boundaries, it might become an issue if we don’t have it worked out. (Participant #10)

Some of the differences are religious and spiritual, and may change the views of the service providers themselves:

There’s also quite a big Muslim community … and getting to understand the details around differences. I learned a lot … how they practice their religion and what is acceptable and unacceptable, how certain people want to be treated or not treated, like for example, when it comes to Muslim men, they do not want to be cared for by Muslim women if it’s not their wife.

Specific communities … being predominantly people from different cultures … patients have really contributed greatly to my understanding. People integrate the ancestors in what they believe and then when a patient passes away the rituals that go with that patient … it’s what’s important to do and what creates meaning, and to honor that, that’s very important. In AAA area [a community in Cape Town which was historically a Black township] … it’s a combination of these more religious Christian elements of community. (Participant #5)

Drawing on the diversity within the SLCH team itself was seen as useful:

There is a couple of African families in BBB area [less affluent community] where we actually ended up bringing in one of our African sisters [nurse] … to help us deal with the patient who was an older lady. We needed someone that understood … patient actually come from the Eastern Cape [a largely rural province from which many African people in Cape Town migrate in search of work]. (Participant #7)

Participants emphasized how respect for different practices may be a way of countering a history of paternalism:

I think it’s enormously important that we have some understanding of each other’s cultures because and I think the history of our country has been very White paternalistically, and in health care particularly. … it’s like the White doctor says, and Western doctor says this. We’re not really understanding where people are coming from. (Participant #4)

Openness to the changing nature of society and the fluidity of cultural positionings was also seen as important:

… to try and understand where people are coming from, and you may not be an expert but just have a little understanding and … be open and ask questions. Don’t presume to be knowledgeable and don’t assume that just because this person [patient] was Muslim that they’re going to be doing all the rituals … because cultures are blended and Westernization creeps in and, certainly with Afro-cultural African Christian traditions. (Participant #4)

### Theme 4: Total pain and empowerment

In social contexts in South Africa, empowerment in many areas of life becomes an important aspect of care:

We have to empower the patients and families, even with the wills – our people [Black community] don’t know this, and we must teach them. Mostly me [the social worker] … meet with the patient and the families. (Participant #8)

Empowerment is important as it is different from area to area, and it depends on the needs of the patient in the areas. With SLCH, working in diverse communities, often the apartheid legacy and the apartheid socialization have impacted how people have been groomed in various situations and social contexts.

## Discussion

A noteworthy feature of our discussions with participants was an underlying narrative of a dramatic shift in a service. This service historically, and with the best of intentions, functioned within and reproduced longstanding power hierarchies. It has developed into a more fluid and diverse service, grappling with complex challenges not commonly seen as central or even distinctive to palliative care work.

Despite there being a change in the service offering of the organization, however, the effects of longstanding disparities are still prevalent, especially in less affluent or marginalized areas of service delivery of the organization. When South Africa became a democracy nearly 30 years ago, there was an expectation and hope of more equal distribution of wealth, services and access. It is clear, unfortunately, that SLCH continues to grapple with the realities of gross inequality.

Total pain is not, as we have noted, a new concept, nor is it one that developed in contexts like South Africa. But for our participants, a very large part of the work is dealing with social pain, often without the support of other services which may be taken for granted in wealthier and more equal societies. For our participants, then, their recognition of the local context of total pain required an approach to care which they recognize as insufficient to meet the total needs of dying people and their families. An added challenge and opportunity is the diversity of understandings of life and dying, with its concomitant burden on and gift to the service provider to decenter their usual way of seeing the world and to learn about different ways of living and dying.

It is certainly the case globally that in palliative patient care, holistic care recognizes care that is comprehensive (Strandberg et al. 2007). The challenges faced by SLCH in the current context are not unique and are increasingly a feature of diversifying societies and, in many cases, resource constraints. The case of South Africa and SLCH, though, indicate that the social challenges that accompany processes of death and dying are not something extra to be added on to appropriate palliative care, but are central to how palliative care needs to be delivered in a complex, unequal, and divided world. For palliative care to take its rightful place globally, the discipline needs to listen to the voices of those who provide care in contexts which, though they may seem marginal to people in wealthier countries, actually reflect the majority world. In this respect, it is not by accident that our participants identified issues of power and empowerment as central to good palliative care – much of what they deal with on an everyday basis has its roots in histories of disempowerment, conquest, and domination.

## Conclusion

The concept of total pain (and hence of total care), as developed by Saunders, remains central to good palliative and hospice care. In this article we have shown how this concept is enlivened and broadened within the context of a South African service. For the field of palliative care to develop, though, we need to know more about palliative care in a wide range of different global contexts, many of which face similar challenges to those in South Africa. The question of empowerment in palliative and hospice care is a question not just of empowerment of service users, but also of the voices of those providing care in newer, and more challenging, contexts. In terms of service delivery by the particular hospice we studied, a broader understanding of total pain than is often thought about may be key to further developing services appropriately. We believe that this may well be the case in other contexts as well.

## Footnotes

1 At SLCH, day hospices are day centers for patients who are not bedridden and who attend 1 day per week, having access to holistic services as well as participating in stimulating activities and outings.

2 South African system that divided every man, woman, and child into 4 racial subdivisions, “African,” “Indian,” “Colored,” and “White.” One might have assumed that this madness would be scrapped when White rule was eliminated in 1994 – or so one might have thought. Yet every South African is still racially classified by law.
